# Fracture resistance of mandibular molar endocrown with different preparation designs: an *in vitro* study

**DOI:** 10.7717/peerj.21801

**Published:** 2026-10-22

**Authors:** Pengiran Muhammad Badi’uzzaman Awang Iskanderdzulkarnein, Noorhayati Raja Mohd, Anas Hakimee Ahmad Ubaidillah, Muhammad Aliff Ikram Noor Zari, Siti Fauzza Ahmad, Eshamsul Sulaiman

**Affiliations:** 1 Department of Restorative Dentistry, Faculty of Dentistry, International Islamic University Malaysia, Kuantan, Pahang, Malaysia; 2 Department of Restorative Dentistry, Faculty of Dentistry, Universiti Malaya, Kuala Lumpur, Malaysia

**Keywords:** Fracture resistance, Endocrown, Mandibular molar, Preparation design

## Abstract

**Background:**

Endocrowns have gained popularity as a conservative option for restoring endodontically treated molars because their success rates are comparable to those of conventional crowns. However, there is limited evidence on the effectiveness of different endocrown marginal designs, particularly concerning their fracture resistance and failure modes under axial and oblique loading. This *in vitro* study aimed to evaluate the fracture resistance and failure modes of three different endocrown marginal designs on mandibular molars and compare them with endocrowns under axial and oblique loading.

**Methods:**

Sixty-four extracted mandibular molars were root-treated and lined with flowable composite (1.5 mm thickness) before being embedded in epoxy resin blocks. The samples were divided into four groups (*n* = 16): three endocrown groups with different preparation designs (butt-joint, 0.8 mm chamfer, and 1.0 mm shoulder) and one group with conventional crowns. Crown preparations were performed with a high-speed handpiece mounted on a modified milling device, targeting a 2.0 mm anatomical occlusal reduction for conventional crowns and a 4.0 mm central chamber depth with a 2.0 mm axial wall height (ferrule) for endocrowns (except the butt-joint design, which had no ferrule preparation). Restorations were fabricated using the Chairside Economical Restoration of Esthetic Ceramics (CEREC) Computer-Aided Design and Computer-Aided Manufacturing (CAD/CAM) system with lithium disilicate (IPS e.max CAD; minimum occlusal thickness: 1.5 mm) as the restorative material and cemented with the RelyX Ultimate dual-cure resin adhesive. After 1,500 thermocycling cycles, the samples were divided into two subgroups (*n* = 8) for axial and oblique loading tests. The fracture resistance was tested using a universal testing machine at a crosshead speed of 0.5 mm/min, applying a load of up to 5,000 N until fracture. Failure modes were examined using a stereomicroscope at 5x magnification and categorized as catastrophic or non-catastrophic. The mean fracture resistance was compared using one-way Analysis of Variance (ANOVA) with Bonferroni *post-hoc* tests, and Fisher’s exact test was used to assess the association between preparation designs and failure modes (*p* < 0.05).

**Results:**

Endocrowns with shoulder margins showed significantly higher fracture resistance (3,042.83 ± 850.76 N) than conventional crowns (1,908.28 ± 635.82 N) under axial loading (*p* = 0.02). No significant differences were found under oblique loading between the preparation designs. The preparation design significantly influenced the failure mode under axial loading (*p* = 0.049). Endocrowns exhibited a higher rate of catastrophic fractures than conventional crowns, regardless of loading direction.

**Conclusions:**

Endocrowns provide a conservative alternative to conventional crowns, offering a higher fracture resistance under axial loading. Further research is needed to evaluate the endocrown performance under oblique loading.

## Introduction

Endodontically treated teeth (ETT) are particularly vulnerable to structural compromise due to caries, trauma, and the invasive nature of endodontic procedures. These conditions can significantly reduce fracture resistance, thereby increasing the risk of fractures under occlusal loading. Research has consistently shown that ETT exhibit lower fracture resistance than vital teeth, a problem further exacerbated by re-endodontic procedures that result in additional loss of root dentin, thereby further compromising tooth integrity (Usta et al., 2023; Almutairi, Abdallah & Genena, 2024). As such, achieving durable coronal restoration is essential for the long-term survival of ETT (Al-Meshal et al., 2025). However, conventional restorative techniques often necessitate substantial removal of the tooth structure, which may not be ideal for teeth that have already been compromised by extensive decay or prior restorations.

In recent years, endocrowns have emerged as viable and conservative alternatives to traditional post-core-supported crowns. Monolithic ceramic restorations, which employ adhesive bonding techniques, are particularly advantageous for molars with severely compromised coronal structures. By incorporating the pulp chamber and cavity margins into their design, endocrowns preserve more of the natural tooth structure, while minimizing invasiveness. This approach provides a favorable fracture resistance profile, making endocrowns particularly suitable for molars that require robust restorations to withstand significant masticatory forces (Popa et al., 2024; Khattab, Ali & Mohammed, 2022).

The extra-coronal height of a restoration acts as a lever arm during loading, with higher conventional crowns creates longer moment arms that increase bending stress at the cervical margin. In contrast, endocrowns utilize a deep intracoronal extension into the pulp chamber (*e.g.*, 4.0 mm depth), which shortens the extra-coronal lever arm and shifts the effective fulcrum apically into the tooth structure. By securing the retention internally rather than relying on tall external walls, endocrowns reduce unfavorable bending moments and improve force distribution under heavy occlusal loads.

Despite the increasing popularity of endocrowns owing to their conservative nature and favorable mechanical properties, the comparative effectiveness of various endocrown marginal designs, particularly concerning fracture resistance and failure modes under axial and oblique loading conditions, remains insufficiently explored. Some studies have proposed that certain design modifications, such as the addition of a ferrule or the use of advanced materials, such as lithium disilicate, could enhance fracture resistance (Ghoul, Abdallah & El-Sheikh, 2020; Chaudhary et al., 2025). However, the extent to which different marginal configurations, including butt joint, chamfer, and shoulder designs, offer substantial improvements over the standard butt joint configuration, especially for mandibular molars, remains unclear. This gap in the literature necessitates a comprehensive *in vitro* study to evaluate the fracture resistance and failure modes associated with different endocrown designs compared with conventional crowns.

Recent research into endocrown preparation designs highlights that margin configurations and internal modifications are critical factors in determining the mechanical behavior, marginal fit, and fracture resistance of restorations in endodontically treated teeth. Specifically, studies have shown that a 20° bevel margin significantly reduces stress concentrations on dentin compared to flat butt joint or shoulder designs, while incorporating retention grooves or increasing cavity depth can further enhance fracture resistance (Zheng et al., 2022; Ghoul et al., 2020). However, there is a trade-off, as deeper and more complex preparations can lead to greater marginal discrepancies (Mostafavi et al., 2022). Regarding fit, shoulder preparations have demonstrated superior marginal and pulpal floor adaptation compared to onlays, though intraradicular extensions may negatively affect this fit (Soliman et al., 2022; Heeter et al., 2025). Additionally, while a one mm ferrule may improve failure loads, its clinical significance is still debated, and horizontal preparations like the chamfer are generally preferred for maximizing the preservation of tooth structure (Einhorn et al., 2017). Collectively, these findings suggest that subtle geometrical adjustments in preparation design—ranging from finish line type to internal wall configuration—are essential for optimizing both the structural integrity and clinical longevity of endocrowns.

Moreover, the existing research exhibits certain biases that may limit the generalizability of the findings. For example, some studies have focused on mandibular premolars rather than molars, where endocrowns are more frequently utilized, potentially leading to misleading conclusions regarding their effectiveness (Thomas et al., 2020). Additionally, several investigations have not adequately accounted for differences in marginal continuity and the distinct geometry of molars *versus* premolars, which are factors that can significantly influence both adhesion and the overall success of endocrowns (Thomas et al., 2020). A systematic review examining the five-year survival rates of endocrowns and conventional crowns reported no significant differences; however, the review’s emphasis on specific materials and designs may have biased the findings, thus limiting their applicability to all endocrown types (Al-Dabbagh, 2021).

To address these gaps in the existing literature, this study aimed to evaluate the fracture resistance and failure modes of three different endocrown marginal designs, specifically butt-joint, chamfer, and shoulder, on mandibular molars, and to compare these outcomes with those of conventional crowns under both axial and oblique loading conditions. The study hypothesized that there is no significant difference in fracture resistance and failure modes between the various endocrown preparation designs and conventional crowns in endodontically treated mandibular molars under these loading conditions.

## Materials & Methods

This *in vitro* experimental study was reviewed and approved by the local Institutional Review Board of University Malaya Medical Ethics Committee (Ref: DF RD2204/0010 (P)). All extracted teeth used in this study were extracted due to orthodontic or periodontal reasoning. Written consent was taken from the patient whose teeth were used in the present study for experimental purposes in accordance with the Declaration of Helsinki (revised in 2013).

### Sample collection and preparation

A total of 232 extracted mandibular molars were collected and disinfected according to ISO/TS 11405:2003 guidelines. The teeth were initially preserved in 0.9% physiological saline solution to prevent desiccation and subsequently disinfected in 0.5% chloramine T solution for one week. After debris and calculi were removed with an ultrasonic scaler, the teeth were stored in distilled water at 4 °C to maintain hydration until further use.

### Sample size calculation

Sixty-four mandibular molars were selected and the sample size was calculated using G*Power software (Version 3.1.9.7) resulting in 64 samples. This calculation provided adequate statistical power to detect significant differences, accounting for a 15% failure rate during the sample preparation.

### Canal preparation and obturation

All samples underwent standardized endodontic treatment using a crown-down technique. Root canals were prepared using ProTaper Next rotary files (Dentsply Sirona, Charlotte, NC, USA), irrigated with 3% sodium hypochlorite and 17% EDTA, and obturated using the continuous wave condensation technique with Protaper Next gutta-percha and an epoxy resin-based sealer (AH Plus Jet; Dentsply Sirona, Charlotte, NC, USA). After obturation, a 1.5 mm layer of bulk-fill flowable composite was applied over the canal orifices to provide a uniform base. The samples were then embedded in epoxy resin blocks (Mirapox 950-230, Miracon Sdn. Bhd, Malaysia) to simulate alveolar bone support, with the resin level standardized at 2.0 mm below the CEJ.

### Sample distribution and crown preparation

The samples were randomly divided into four groups (*n* = 16) according to the type of restoration (Table 1): conventional crowns (Group T) and endocrowns with three different marginal designs: butt joint (Group B), chamfer (Group C), and shoulder (Group S). Tooth preparations were standardized using a high-speed handpiece mounted on a modified milling machine (BF-2; Bredent GmbH, Germany) to ensure precision and consistency (Fig. 1).

**Table 1:** Group distribution, schematic diagram, and tooth preparation design for each main group.

Total sample size	*N* = 64			
Main group and size	Lithium disilicate endocrown with a butt-joint margin	Lithium disilicate endocrown with a chamfer margin	Lithium disilicate endocrown with a shoulder margin	Conventional lithium disilicate crown (control)
	(*n* = 16)	(*n* = 16)	(*n* = 16)	(*n* = 16)
Schematic diagram	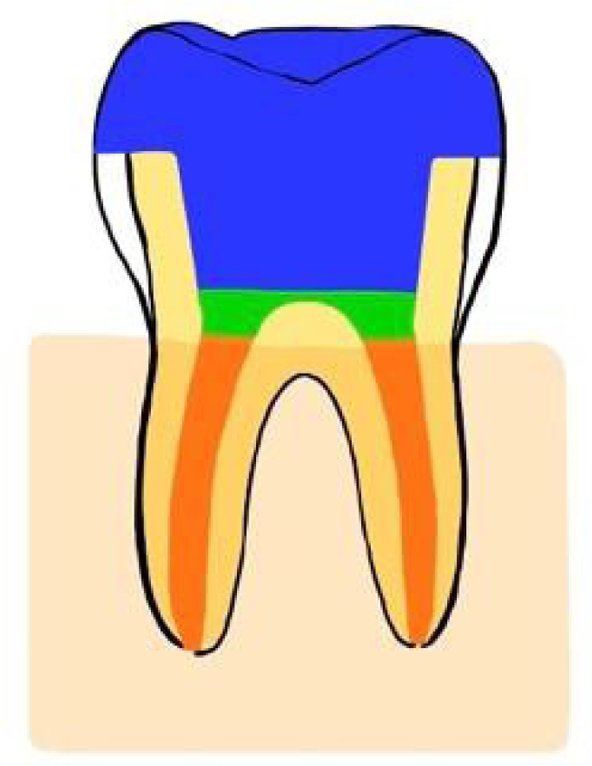	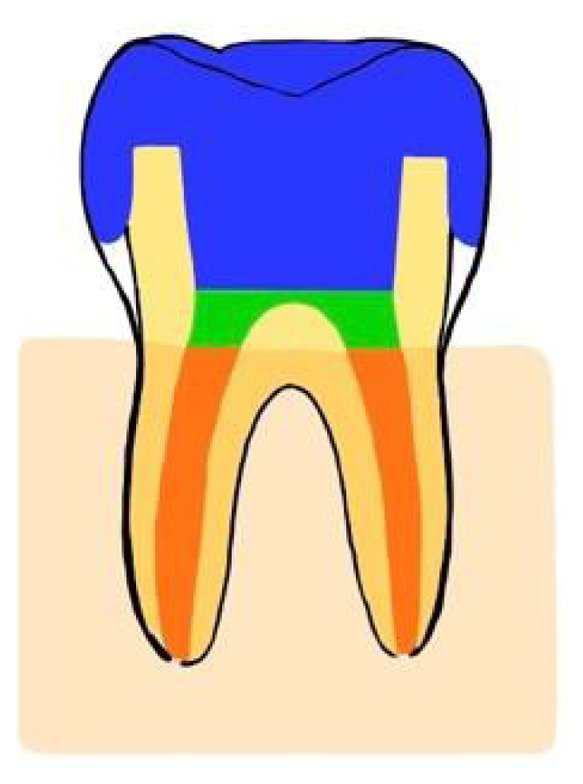	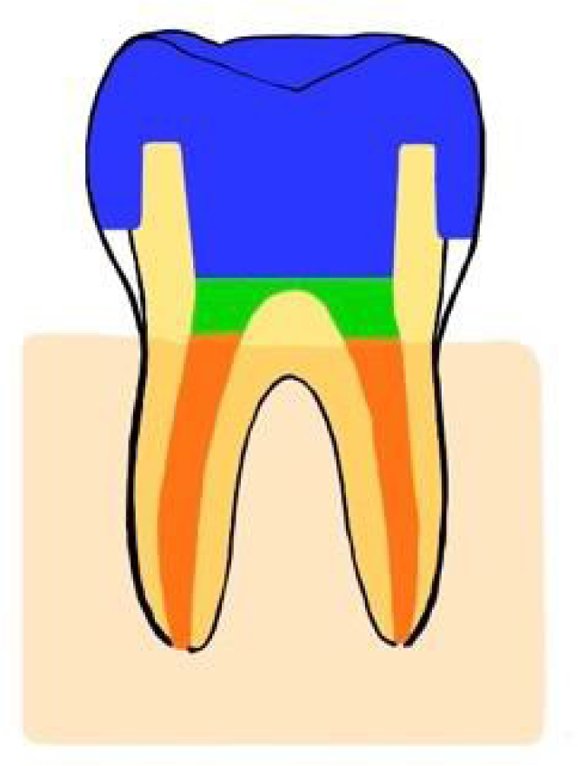	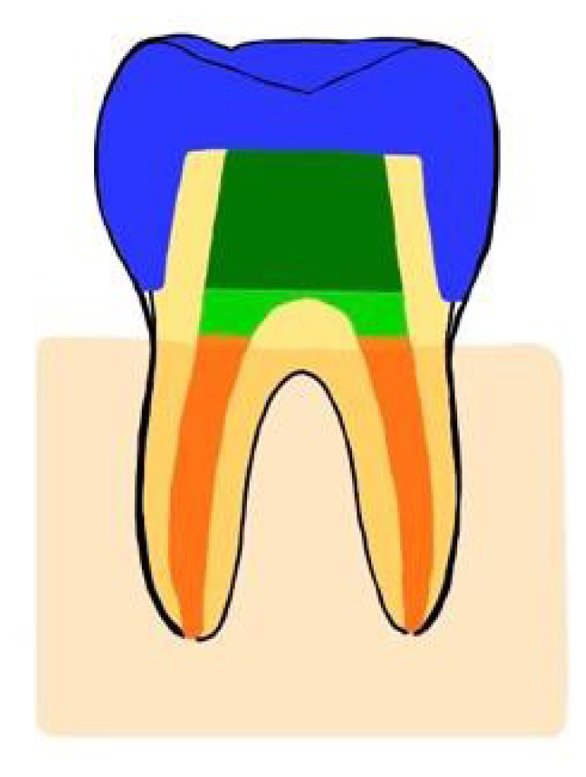
Main group coding	Group B	Group C	Group S	Group T
Central chamber depth	4.0 mm from central fossa	4.0 mm from central fossa	4.0 mm from central fossa	–
Degree of taper for central chamber wall (internal ferrule)	10° of total divergence angle	10° of total divergence angle	10° of total divergence angle	–
Margin type	Butt-joint or flat top margin	0.8 mm chamfer margin	1.0 mm rounded shoulder margin	1.0 mm rounded shoulder margin
Margin termination or axial height	–	2.0 mm axial height measured after flat occlusal reduction	2.0 mm axial height measured after flat occlusal reduction	Minimum of 2.0 mm axial height measured after anatomical occlusal reduction at distal marginal ridge
Degree of taper for axial wall (external ferrule)	–	12° of total convergence angle	12° of total convergence angle	12° of total convergence angle

**Figure 1:**
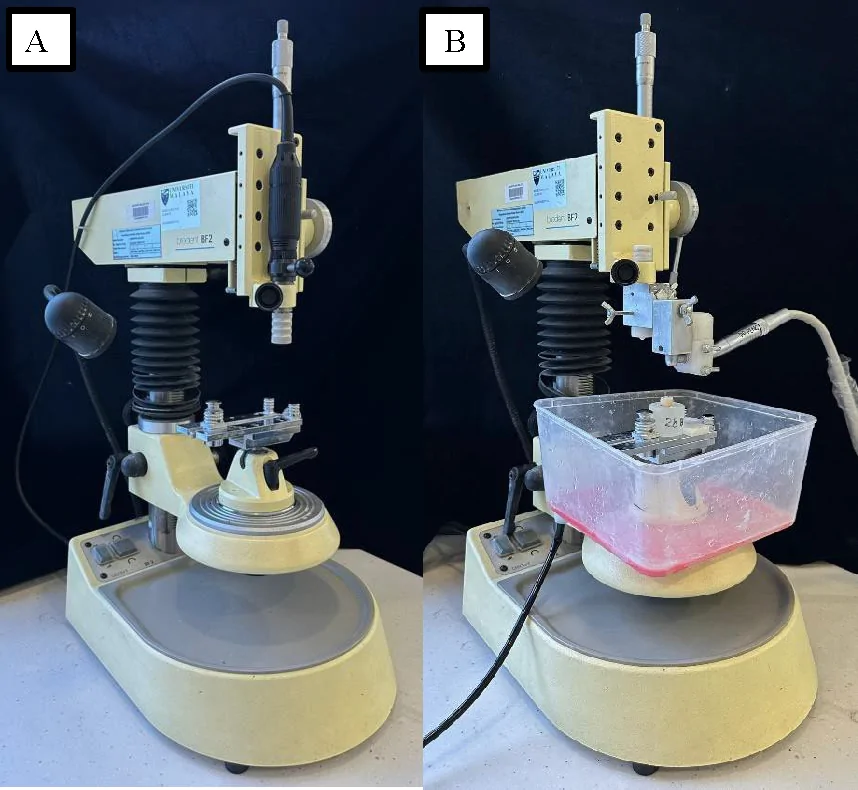
Milling machine (Bredent GmBH, Germany) modification with a high-speed contra-angle handpiece (T3 Racer, Sirona, Germany) through a custom-made aluminium adapter. (A) Before modification and (B) After modification.

### Endocrown with a butt-joint margin (Group B) preparation

The preparation of the endocrown with a butt-joint margin (Group B) followed a highly standardized protocol utilizing a modified milling machine (Bredent BF-2) equipped with a high-speed contra-angle handpiece and specialized diamond burs to ensure mechanical precision and parallelism. After the removal of temporary restorations, a wheel-shaped diamond bur was used for occlusal flattening to establish a uniform reference point, while the pulpal floor was leveled to create a standardized central chamber depth of 4.0 mm, verified by a digital depth gauge. This leveling process simultaneously established a 10° total divergence angle for the chamber walls, effectively forming an internal ferrule (Fig. 2). To finalize the preparation for CAD/CAM fabrication, sharp internal line angles were systematically rounded using a specialized superfine grit diamond bur.

**Figure 2:**
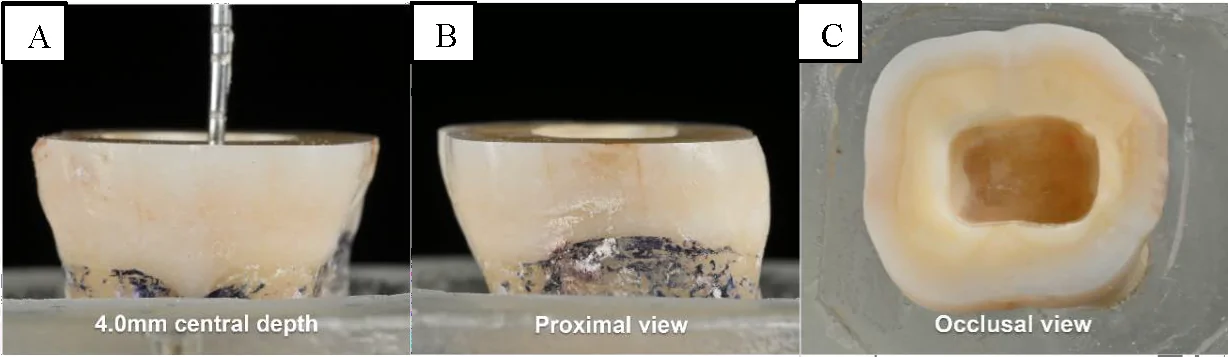
The final outcomes of tooth preparation for an endocrown with a butt-joint margin (Group B) from various views. (A) Labial view with 4.0 mm central depth, (B) Proximal view and (C) Occlusal view.

### Endocrown with a chamfer margin (Group C) preparation

The preparation of the endocrown with a chamfer margin (Group C) followed the foundational procedures used for the butt-joint design with additional steps to achieve the specific finish line geometry. Using a tapered round-end medium grit diamond bur equipped with a guide pin to serve as a horizontal stopper (998-026M-FG, NTI-Kahla GmbH, Germany), the axial height was precisely established at 2.0 mm with a consistent 0.8 mm chamfer depth around the margin (Fig. 3). This standardized axial reduction protocol was also implemented for Groups S and T to ensure methodological uniformity across the study. Finally, to eliminate areas of concentrated stress and optimize the tooth for CAD/CAM fabrication, all sharp internal and external line angles were systematically rounded using a specialized superfine grit diamond bur.

**Figure 3:**
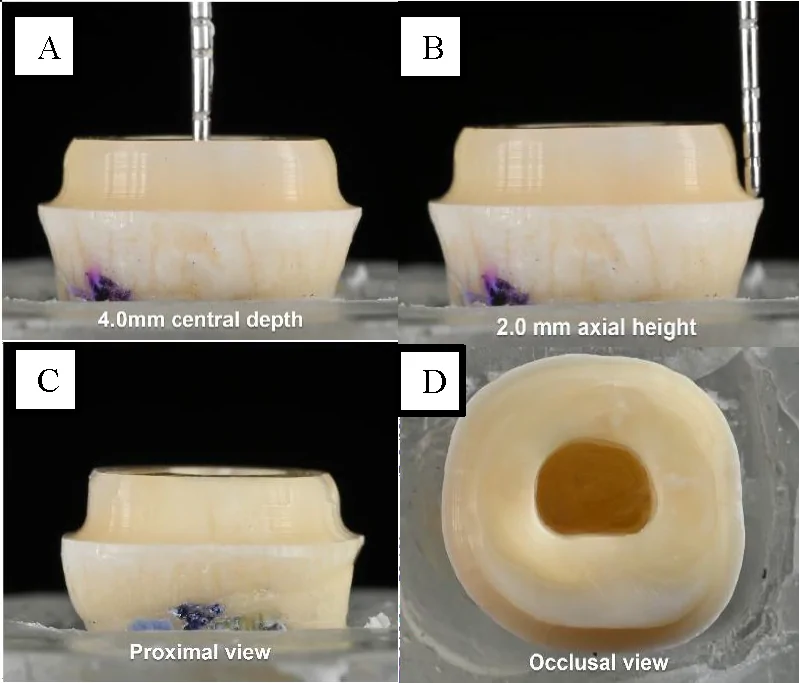
The final outcomes of tooth preparation for endocrown with a 0.8 mm chamfer margin (Group C) from various view. (A) Labial view with 4.0 mm central depth, (B) Labial view with 2.0 mm axial height, (C) Proximal view and (D) Occlusal view.

### Endocrown with a shoulder margin (Group S) preparation

The preparation of the endocrown with a shoulder margin (Group S) built upon the protocols for the butt-joint and chamfer designs, involving the further refinement of the 0.8 mm chamfer into a 1.0 mm rounded shoulder margin. This transition was achieved using a tapered round-end superfine grit diamond bur, with measurements verified by a periodontal probe to ensure precision. To optimize the preparation for CAD/CAM fabrication and prevent stress concentration, all sharp internal and external line angles were systematically rounded using a specialized superfine grit diamond bur. These steps finalized the design, which maintained a standardized 4.0 mm central chamber depth and 2.0 mm axial height, as illustrated in the final preparation outcomes (Fig. 4).

**Figure 4:**
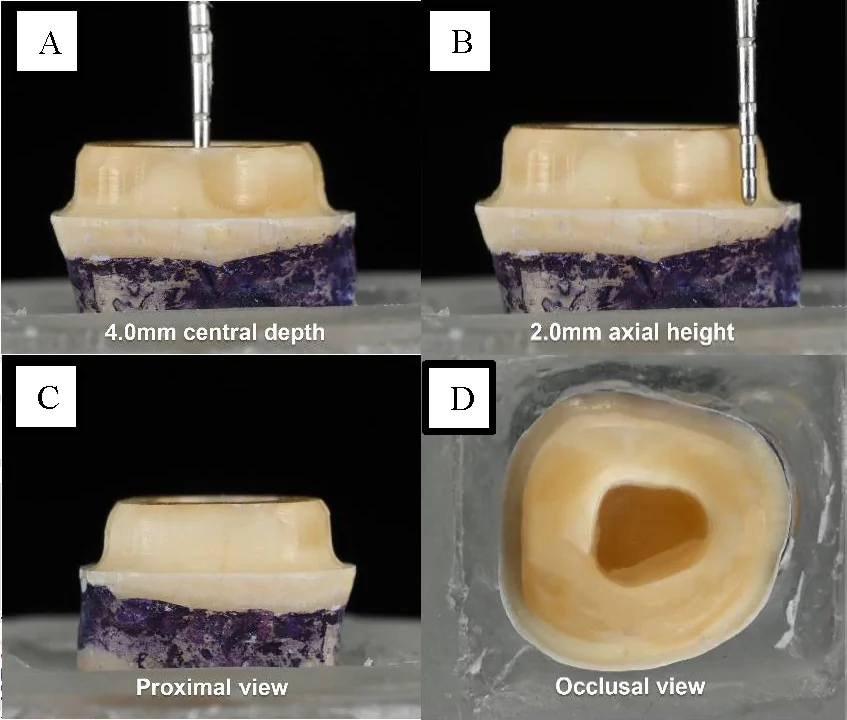
The final outcomes of tooth preparation for endocrown with a 1.0 mm shoulder margin (Group S) from various view. (A) Labial view with 4.0 mm central depth, (B) Labial view with 2.0 mm axial height, (C) Proximal view and (D) Occlusal view.

### Conventional crown (Group T) preparation

The preparation of the conventional crown control group (Group T) involved a standardized protocol beginning with the permanent sealing of the access opening using Filtek Z350 XT packable composite following a meticulous adhesive procedure. The clinical preparation consisted of a 2.0 mm anatomical occlusal reduction with functional cusps beveled at a 45-degree angle, alongside an axial reduction height of 2.0 mm (Fig. 5). A distinct 1.0 mm rounded shoulder margin was achieved through refined axial deepening and verified using a periodontal probe, while all sharp line angles were systematically rounded to facilitate CAD/CAM fabrication and optimize the final restoration’s fit.

**Figure 5:**
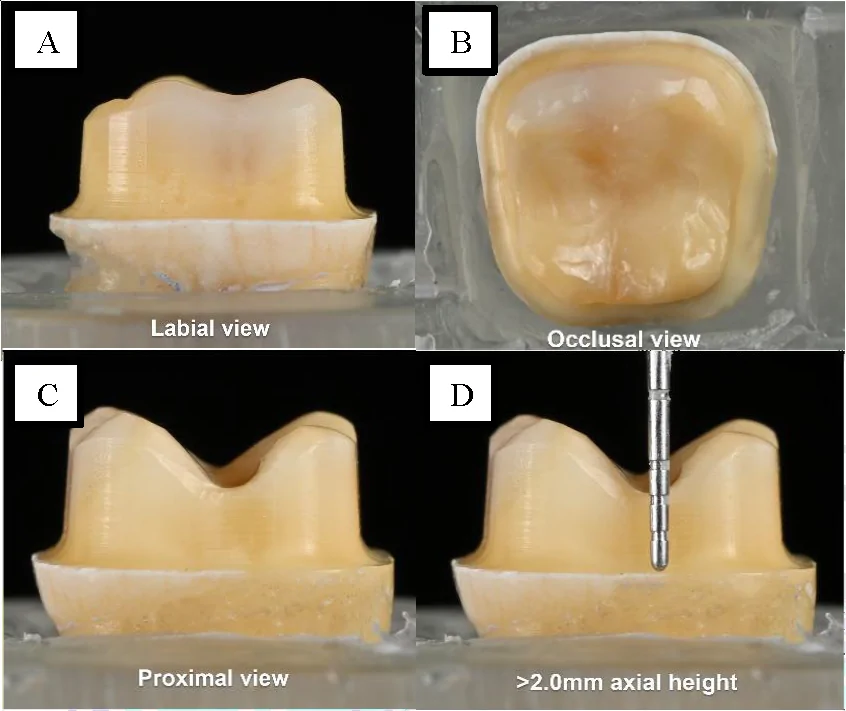
The final outcomes of conventional crown tooth preparation (Group T) from various view. (A) Labial view, (B) Occlusal view, (C) Proximal view and (D) Proximal view with less than 2.0 mm axial height .

### Fabrication of restoration

All restorations were fabricated using the CEREC chairside CAD/CAM system (Dentsply Sirona, Charlotte, NC, USA), enabling the precise design and milling of lithium disilicate blocks (IPS e.max CAD, Ivoclar). Each tooth was scanned in a custom-made jig to ensure consistent positioning and size, and the restorations were designed with minimum thicknesses of 0.8 mm radially and 1.5 mm occlusally (Fig. 6). Following milling, crystallization, and finishing, each restoration was attempted to evaluate fit and marginal adaptation. The margin thickness, 0.8 mm was verified using caliper for the standardization to of the restoration’s thickness after milling process. Later, the intaglio surfaces were etched with 5% hydrofluoric acid for 20 s, rinsed, dried, and treated with silane to enhance the bonding. The prepared teeth were etched with 32% phosphoric acid, rinsed, dried, and treated with a Universal Adhesive bonding agent (3M™ Scotchbond Universal Adhesive; 3M ESPE, USA).

**Figure 6:**
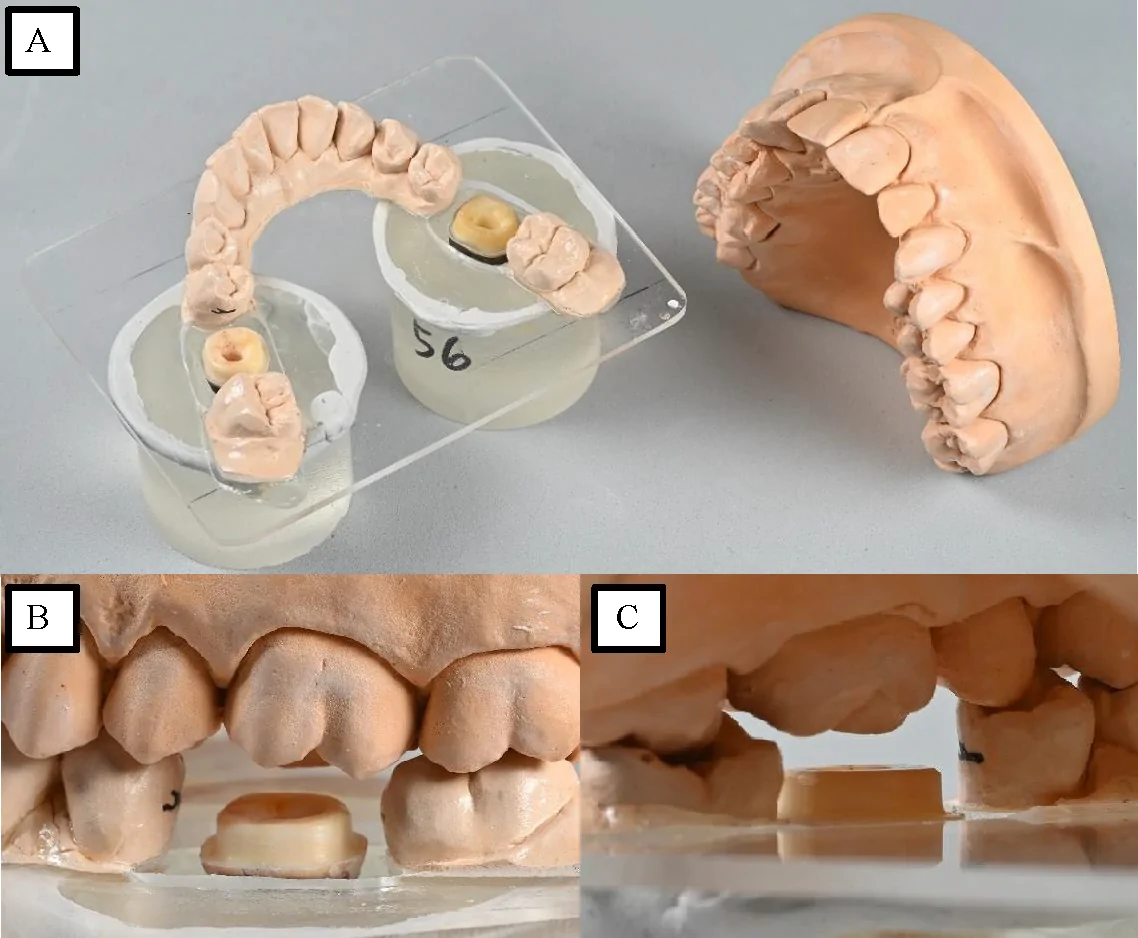
Preparation prior to initiation of the CAD/CAM process. (A) Tooth samples were securely affixed in a custom-made scanning jig representing a full-mouth dentition, constructed from a stone model and a transparent acrylic sheet; (B) mesiodistal view of the model; and (C) vertical view of the model to access the restorative space during a digital scanning procedure.

Cementation was performed using RelyX Ultimate dual-cure resin adhesive (3M ESPE, USA), with the restorations seated using firm finger pressure, according to the manufacturer’s instructions. Using the tack curing function on the 3M™ Elipar™ DeepCure-L light curing unit (3M ESPE, USA), the restoration was tack-cured for 1–2 s per surface. The excess cement was removed after tack curing, followed by final curing for 20 s per surface, to ensure complete polymerization of the resin cement. Finally, finishing and polishing were completed using a lithium disilicate polishing kit (Diapol^®^ RA, Eve Ernst Vetter GmbH, Germany).

To simulate oral conditions, all samples underwent 1,500 thermocycles between 5 and 55 °C, with a dwell time of 20 s per cycle, following the ISO/TS 11405:2003 guidelines.

### Fracture resistance test

For the experimental phase, each main group was randomly subdivided into two distinct subgroups of eight specimens each (*n* = 8) to undergo either axial loading (TA, BA, CA, SA) or oblique loading (TO, BO, CO, SO) tests. Fracture resistance was measured using a universal testing machine (Autograph AGS-X, Shimadzu Corp.) equipped with Trapezium X software, where settings were standardized for compression testing with a crosshead speed of 0.5 mm/minute and a maximum force cap of 5000 N. Forces were applied using a 3.0 mm diameter stainless-steel loading rod affixed to the upper mobile component of the machine and the specimens were defined as having glass material and an area shape.

### Axial loading test

The samples undergoing the axial loading test were from groups TA, BA, CA, and SA. Each sample was placed in a standard metal holder of the universal testing machine, aligning the occlusal table parallel to the floor. The load was specifically applied to the central fossa of the tooth samples, replicating the contact point during Maximum Intercuspal Position (MIP). Once all positions were secured, the testing began at a 0 reading, and the compression test proceeded until the specimen reached its breaking point. The maximum force required for material fracture, known as fracture resistance, was quantified in Newtons (N) and systematically recorded in the Trapezium X software.

### Oblique loading test

The samples subjected to the oblique loading test were drawn from groups TO, BO, CO, and SO. Each sample was positioned in a custom-metal jig (Faculty of Engineering, University Malaya), aligning the long axis of the tooth at a 135° angle to the direction of the load. The load was specifically directed to the buccal slope between the mesio-lingual and disto-lingual cusps, simulating the contact point at the working side during lateral excursion. After securing all positions, the test initiated at a 0 reading, and the compression test continued until the specimen reached its breaking point. The maximum force needed for material fracture, denoted as fracture resistance, was measured in Newtons (N) and systematically recorded in the Trapezium X software.

### Determination of mode of failure

Failure modes were assessed using a stereomicroscope (U-CMAD3, Olympus, Japan) at 5x magnification, categorizing fractures as catastrophic or non-catastrophic (Abdulrazzak et al., 2014; Alarami, Sulaiman & Al-Haddad, 2017; Sulaiman et al., 2018).

#### Non-catastrophic fracture

This category includes failures occurring above the level of the epoxy resin block, which serves as a simulation of the bone level. Non-catastrophic failures are considered favourable, allowing for the restoration of the tooth as depicted in Fig. 7. The classification include:

**Figure 7:**
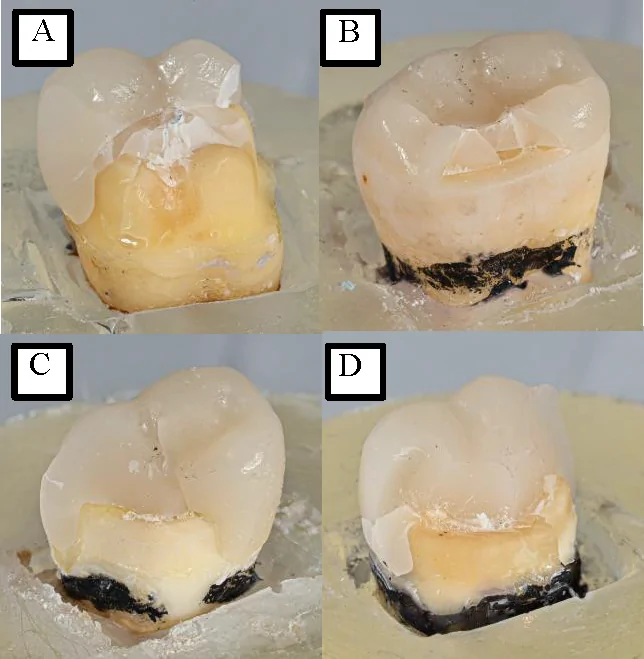
Example of non-catastrophic fracture. (A) Conventional crown, (B) endocrown with butt-joint margin, (C) endocrown with chamfer margin, and (D) endocrown with shoulder margin.

Class I: Complete debonding of the prosthesis

Class II: Partial debonding of the crown

Class III: Fracture of the crown-tooth complex above the epoxy level.

#### Catastrophic fracture

In contrast, catastrophic failures involve fractures below the level of the epoxy resin block, making the tooth non-restorable as in Fig. 8. The classification include:

**Figure 8:**
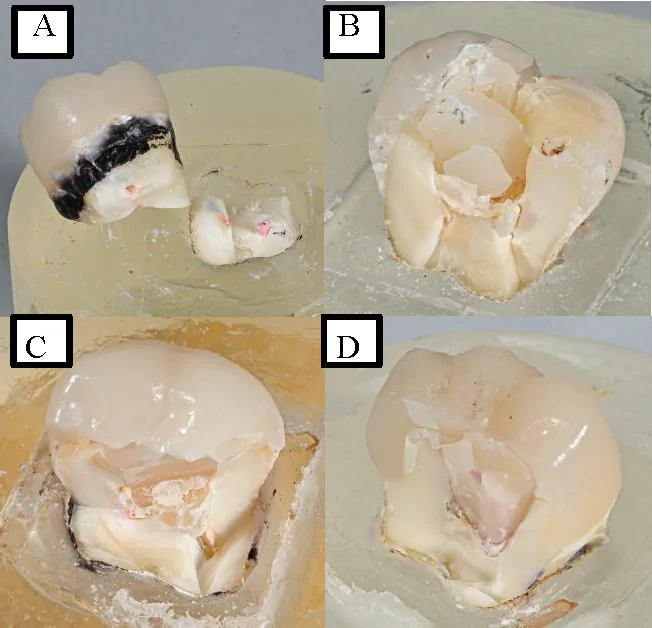
Example of catastrophic fracture. (A) Conventional crown, (B) endocrown with butt-joint margin, (C) endocrown with chamfer margin, and (D) endocrown with shoulder margin.

Class IV: Fracture of the crown-tooth complex below the epoxy level

Class V: Crack below the epoxy level

Class VI: Vertical root fracture

### Data collection and statistical analysis

The fracture resistance data were analyzed using SPSS version 29 (IBM, USA). Descriptive statistics were calculated for each group. One-way analysis of variance (ANOVA) was used to compare mean fracture resistance among the groups, with Bonferroni *post-hoc* tests for multiple comparisons when significant differences were found. The association between the preparation design and failure mode was assessed using Fisher’s exact test, with a significance level set at *p* < 0.05. A flowchart for this method is shown in Fig. 9.

## Results

### Fracture resistance

The study revealed that preparation design significantly influences fracture resistance under axial loading (*p* = 0.028). The endocrown with a shoulder margin (SA) exhibited the highest mean fracture resistance (3,042.83 ± 850.76 N), while the conventional crown (TA) demonstrated the lowest (1,908.28 ± 635.82 N) (Table 2). Normality tests (Kolmogorov–Smirnov and Shapiro–Wilk) confirmed normal distributions for all groups (*p* > 0.05), supporting the use of parametric tests. *Post-hoc* multiple comparisons using the Bonferroni test confirmed a statistically significant difference specifically between the TA and SA groups (*p* = 0.020). No significant differences were observed between the other axial preparation designs (*p* > 0.05).

In contrast, oblique loading conditions showed no statistically significant difference in fracture resistance among the various designs (*p* = 0.193) (Table 2). Under these conditions, the mean fracture resistance ranged from 1,674.32 ± 753.00 N for conventional crowns (TO) to 1,198.96 ± 361.51 N for the chamfer margin design (CO).

**Figure 9:**
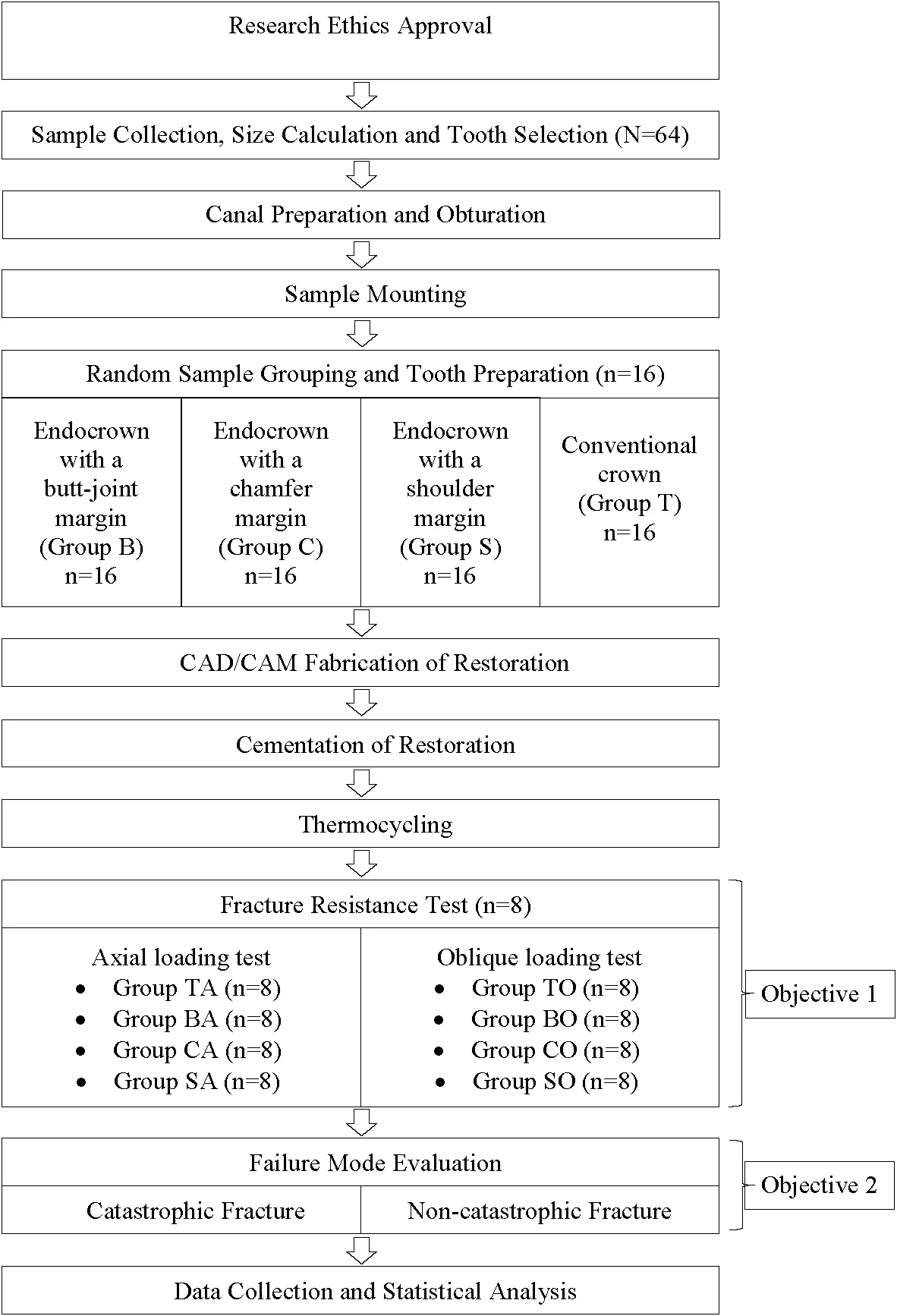
Flowchart summarizing the methodology used in this study.

**Table 2:** Comparison of mean fracture resistance and *post-hoc* analysis.

**Loading condition**	**Preparation design**	**Mean fracture resistance (N) ± SD**	**ANOVA *p*-value**	***Post-hoc* comparison (Bonferroni *p*-value)**
Axial loading	Endocrown with shoulder margin (SA)	3,042.83 ± 850.76	**0.028** ^*^	**TA *vs.* SA (0.020)** ^*^
	Endocrown with butt-joint margin (BA)	2,486.16 ± 611.28		TA *vs.* BA (0.685)
	Endocrown with chamfer margin (CA)	2,318.53 ± 712.43		TA *vs.* CA (1.000)
	Conventional crown (TA)	1,908.28 ± 635.82		–
Oblique loading	Conventional crown (TO)	1,674.32 ± 753.00	0.193	N/A
	Endocrown with butt-joint margin (BO)	1,583.52 ± 550.19		N/A
	Endocrown with shoulder margin (SO)	1,226.88 ± 351.92		N/A
	Endocrown with chamfer margin (CO)	1,198.96 ± 361.51		N/A

**Notes.**

* Significant at *p* < 0.05.

SD Standard Deviation N/A *Post-hoc* tests were not performed for oblique loading as the ANOVA result was not statistically significant

Bold indicates the statistically significant difference between the TA and SA groups (*p* < 0.020) in *post-hoc* multiple comparisons using the Bonferroni test.

### Mode of failure

Table 3 shows that under axial loading, endocrowns, regardless of design, had a high rate of catastrophic fractures (87.5%), while conventional crowns (TA) had a lower rate (37.5%). Fisher’s Exact test revealed a significant association between preparation design and failure mode (*p* = 0.049). For oblique loading (Table 3), both endocrowns with a shoulder margin (SO) and conventional crowns (TO) showed high rates of catastrophic fractures (87.5%), whereas endocrowns with a butt joint (BO) had the lowest rate (50.0%). Fisher’s exact test indicated no significant association between preparation design and failure mode (*p* = 0.426). Overall (Table 4), Group S had the highest rate of catastrophic fractures (87.5%), whereas Group T had the lowest (62.5%). Fisher’s exact test found no significant association between preparation design and failure mode across all conditions (*p* = 0.412).

**Table 3:** Types of fractures and their association with different preparation designs under axial and oblique loading (*N* = 32).

Loading condition	Preparation design	Sample size (n)	Catastrophic	Non catastrophic	*p*-value
			**Fractures *n* (%)**		
Axial Loading	Conventional crown (TA)	8	3 (37.5)	5 (62.5)	0.049^*^
	Endocrown with butt-joint margin (BA)	8	7 (87.5)	1 (12.5)	
	Endocrown with chamfer margin (CA)	8	7 (87.5)	1 (12.5)	
	Endocrown with shoulder margin (SA)	8	7 (87.5)	1 (12.5)	
Oblique	Conventional crown (TO)	8	7 (87.5)	1 (12.5)	0.426
	Endocrown with butt-joint margin (BO)	8	4 (50.0)	4 (50.0)	
	Endocrown with chamfer margin (CO)	8	6 (75.0)	2 (25.0)	
	Endocrown with shoulder margin (SO)	8	7 (87.5)	1 (12.5)	

**Notes.**

* Association test was done with Fisher’s Exact Test.

Significant at *p* < 0.05.

**Table 4:** Types of fractures and their association with different preparation designs under overall loading (*N* = 32).

Preparation design	Catastrophic fractures (n)	Catastrophic fractures (%)	Non-catastrophic fractures (n)	Non-catastrophic fractures (%)	*p*-value
Endocrown with shoulder margin (S)	14	87.50	2	12.50	0.412
Endocrown with chamfer margin (C)	13	81.25	3	18.75	
Endocrown with butt-joint margin (B)	11	68.75	5	31.25	
Conventional crown (T)	10	62.50	6	37.50	

**Notes.**

Association test was done with Fisher’s Exact Test.

Significant at *p* < 0.05.

## Discussion

This study compared the fracture resistance and failure modes of different endocrown designs and conventional crowns under axial and oblique loadings. These findings partially reject the null hypothesis, indicating significant differences in fracture resistance and failure modes among the various lithium disilicate endocrown margin designs.

A primary finding was the statistically significant superior fracture resistance of endocrowns with a shoulder margin (SA) compared to conventional lithium disilicate crowns (TA) under axial loading (3,042.83 N *vs.* 1,908.28 N). This enhanced performance is attributed to the shoulder margin’s ability to increase marginal thickness and distribute stress more effectively across the underlying tooth structure. This approach, which is consistent with the principles of stress distribution in dental restorations, suggests a more uniform stress distribution, leading to a reduced of fracture. These results align with previous research indicating that modified designs featuring thicker occlusal sections and shoulder finish lines contribute to greater structural integrity (Alghafees & Rayyan, 2024; Fayed, Azer & Aboelhassan, 2025). The data clearly indicate that endocrowns with a shoulder margin offer superior structural integrity under stress, reinforcing the importance of considering the design elements in dental restorations to enhance their performance and longevity. Haralur et al. (2020) supported these findings by showing increased strength in molar endocrowns with thicker occlusal sections in lithium disilicate ceramics, with the mean fracture resistance increasing from 2,863.62 N to 3,777.28 N with increased thickness. El Ghoul et al. (2019) reported a higher fracture resistance (2,914 N) in lithium disilicate endocrowns under axial loading, than those under lateral loading, suggesting the importance of load direction in the assessment of crown durability emphasizing the robustness of the modified designs. Interestingly, no significant differences were found between the butt-joint (BA) and chamfer (CA) designs, suggesting that the specific geometry of the 1.0 mm shoulder margin is the critical factor for enhancing resistance to axial forces (Altier et al., 2018; Taha et al., 2018; Einhorn et al., 2017).

In contrast to axial results, no statistically significant differences in fracture resistance were found under oblique loading (*p* = 0.193). While the conventional crown (TO) exhibited the highest mean resistance (1,674.32 N) and the chamfer design (CO) the lowest (1,198.96 N), the lack of significance suggests that margin design has a reduced influence when subjected to oblique loading forces. Under these conditions, factors such as material composition and the specific vector of the applied load may play a more dominant role in determining the restoration’s durability than the preparation geometry itself. Margin design appears to be an important element determining fracture resistance under axial loading, possibly due to the direct transmission of force along the preparation geometry, however the stress distributions are more complex under oblique loading, therefore the margin design becomes less relevant. Conversely, factors such as restorative material properties (strength, modulus, toughness), cementation quality, and the direction and magnitude of applied forces are crucial in determining the durability of the endocrown (Altier et al., 2018; Güngör et al., 2017; Gresnigt et al., 2016). This highlights the need for comprehensive treatment planning, which considers the occlusal dynamics and material selection instead of depending exclusively on the design of the margin preparation to improve the resistance to fracture under physiological settings.

Categorizing failure modes simply as catastrophic *vs.* non-catastrophic lacks scientific depth for brittle ceramic restorations. The Academy of Dental Materials (ADM) guidance on fractography (Scherrer et al., 2017), the authors should evaluate the precise origin of the failure. It is critical to distinguish whether failures initiated as radial cracks originating from tensile stresses at the cementation/preparation interface or as cone cracks originating from contact damage on the occlusal surface. Identifying the exact starting point of the crack is crucial to predicting and understanding true material behavior under stress.

Failure mode analysis revealed that, despite their high fracture resistance, shoulder-margin endocrowns were significantly more prone to catastrophic, non-restorable fractures (87.5%) than conventional endocrowns (37.5%) under axial loading (*p* = 0.049). This pattern suggests that the high rigidity and elastic modulus of lithium disilicate, combined with deep pulpal extensions, may concentrate stress in weaker radicular areas, leading to terminal failures (Jafari et al., 2024; Fraga et al., 2025). This is consistent with theories that thicker restorations are more likely to develop deep cone cracks rather than restorable radial cracks (Jafari et al., 2024; Souza et al., 2022). The significant association established between the design of the axial loading group and the failure mode (*p* = 0.049), as indicated by Fisher’s exact test, emphasizes the importance of design considerations in the failure predictions of dental restorations.

Under oblique loading, the SO and TO groups exhibited high rates of catastrophic fractures (87.5%). In contrast, the butt-joint design (BO) showed the lowest rate of catastrophic failure (50.0%), suggesting it may offer greater robustness or reparability when subjected to lateral masticatory forces. Overall, the conventional crown group (T) maintained the lowest frequency of catastrophic outcomes across all conditions (62.5%), highlighting its continued value in protecting the remaining tooth structure from terminal fracture. This finding aligns with previous research that reported higher catastrophic failure rates in endocrowns, especially in those subjected to lateral loads (Kontakou Zoniou et al., 2025; Lenz, Bacchi & Della Bona, 2023). It has been noted that endocrowns without a ferrule design might lack adequate support, increasing the likelihood of catastrophic failure under stress (Huang et al., 2023). However, no significant association was found between restoration design and failure mode under oblique loading (*p* = 0.426), suggesting that the design may not be a decisive factor in the failure mode under oblique loading rather other factors such as material properties and tooth geometry might play more significant role in determining failure patterns under lateral forces. Overall, this study highlights the critical impact of design factors on the failure modes of dental restoration under axial loading. The high rate of catastrophic fractures observed in endocrowns raises significant considerations for the design and material selection in dental restorations, potentially guiding future improvements in this field.

Addressing the gap in the field, these results suggest a complex relationship between endocrown design and failure mode, which is not solely dictated by the design and occlusal thickness but may involve the utilization of the pulp chamber and the subsequent management of mechanical stress. In present study, for the conventional crown preparations, the pulp chamber is permanently sealed with a resin core to create a solid, stabilized foundation that distributes masticatory forces across the external axial walls. Conversely, the mechanical behavior of the endocrown is heavily influenced by the intracoronal support provided by its 4.0 mm deep intraradicular extension. In this study, the monolithic lithium disilicate (IPS e.max CAD) restoration essentially creates a core region with a high elastic modulus within the pulp chamber. Existing literature suggests that such high-modulus reinforcement in the core significantly improves the overall fracture resistance of the restoration, a trend reflected in our finding that the shoulder-margin endocrown (SA) achieved a statistically superior axial resistance (3,042.83 N) compared to conventional crowns. While this rigid intracoronal support enhances the restoration’s ability to withstand high loads, it also plays a critical role in stress distribution; the high rigidity of the material in this central region may focus stresses toward the radicular dentin, potentially explaining the higher incidence of catastrophic fractures (87.5%) observed compared to the lower rates seen in conventional restorations utilizing more resilient resin-core foundations (Lin, Lin & Zheng, 2020; He et al., 2021). This leads to a future direction in which further studies might explore the influence of varying chamber depths on fracture resistance. For instance, testing endocrowns with a shallower 2.0 mm chamber depth, as opposed to the 4.0 mm depth used in this study, could provide insights into achieving more favorable outcomes.

It is important to emphasize that conventional crowns and endocrowns serve distinct clinical indications and are not intended to compete for the same treatment scenario. Conventional crowns (Group T) are the treatment of choice when there is sufficient natural tooth structure to support a standard 2.0 mm axial preparation without necessitating the use of the pulp chamber for retention. Conversely, endocrowns are indicated for severely compromised endodontically treated teeth where extensive loss of coronal structure makes the pulp chamber the only viable source of internal support, often as an alternative to post-and-core assemblies (Papalexopoulos, Samartzi & Sarafianou, 2021). The comparison in this study is intended to provide a biomechanical benchmark rather than to suggest that a clinician should aggressively reduce intact structure for an endocrown. In fact, our results reinforce a conservative approach; while the shoulder-margin endocrown (SA) offered superior axial resistance, the conventional crown (T) maintained the lowest frequency of catastrophic fractures (62.5%). This confirms that a conventional crown remains the more conservative and predictable option for long-term reparability whenever enough tooth structure is available to support it (Fraga et al., 2025; Naik et al., 2025).

The sample size of eight specimens per subgroup is justified by the study’s ability to achieve sufficient statistical power to identify significant mechanical differences and failure associations. Despite the group size, the data was robust enough to detect a statistically significant difference in fracture resistance between shoulder endocrowns (SA) and conventional crowns (TA) under axial loading (*p* = 0.020), as well as a significant association between preparation design and failure mode (*p* = 0.049). The reliability of these results is further supported by the fact that all groups met the rigorous requirements for parametric testing, with normality confirmed *via* Kolmogorov–Smirnov and Shapiro–Wilk tests (*p* > 0.05) and homogeneity of variance established. This statistical stability was achieved through high-precision standardization, including the use of CEREC CAD/CAM technology for precise milling, custom-made jigs for consistent tooth positioning, and adherence to ISO/TS 11405:2003 guidelines for thermocycling. By minimizing experimental variability through these controlled protocols, the study provides a statistically valid foundation for evaluating preparation designs in a field where a current lack of standardization often limits direct quantitative comparisons.

While present study only focusing on endocrown restoration alone, the existing evidence already suggests that endocrowns perform equally or superiorly to post-core restorations, warranting further direct comparisons through *in vitro*, finite element, and clinical studies to refine restorative guidelines for compromised teeth (Lenz, Bacchi & Della Bona, 2023; Kontakou Zoniou et al., 2025; Qamar et al., 2023). Currrent research emphasizes that comparative studies for endodontically treated teeth (ETT) should prioritize head-to-head evaluations between endocrowns and post-and-core assemblies, as these are the primary clinical restorative choices for teeth with substantial coronal damage. Besides that, this focus is more clinically relevant than comparisons with conventional crowns alone, as traditional crowns typically require sufficient natural tooth structure and are often unsuitable for severely damaged ETT without intraradicular support. Because endocrowns and post-core systems exhibit significantly different biomechanical behaviors and failure patterns, examining their contrasting performances is essential for optimizing clinical decision-making. Consequently, next studies focusing on direct comparisons between post-core systems and endocrowns will yield greater insights for the progression of restorative dentistry.

Despite these findings, it is worth mentioning that this study comes with limitations. Firstly, the *in vitro* nature of this study does not fully replicate the complex conditions of the oral environment, such as the presence of saliva, thermal fluctuations, and variations in chewing patterns. Therefore, future studies with larger sample sizes and more clinically relevant conditions are required to validate these results. Secondly, this focus on both axial and oblique loading only. The variable rates of catastrophic fractures across different designs under such forces emphasize the need for more comprehensive studies. Future studies should focus on evaluating the impact of various design factors under different loading conditions, including a deeper investigation of the relationship between material properties and failure modes. Thirdly, present study solely focuses on the static loading which might not provide the real clinical performance. Thus, by incorporating the dynamic loading tests that simulate the complex forces of chewing and other oral activities for future research may provide a more realistic assessment of how endocrowns withstand everyday use. Fourthly, examining the effect of the force application vector in more detail could provide valuable insights, potentially leading to innovative design modifications for improved performance under real-world masticatory conditions. Concisely, while this study contributes to a better understanding of the failure modes of dental restorations, it also opens the door to future research aimed at optimizing endocrown designs for both durability and reparability, particularly considering the implications of aging on the specimens and the real-world conditions they must withstand.

## Conclusions

Within the limitations of this study, the following conclusions were drawn:

1. Endocrowns with a shoulder margin exhibited the highest fracture resistance under axial loading, while no significant differences were found under oblique loading, indicating that the marginal design is more critical for resistance to axial forces than oblique forces.
2. Although shoulder-margin endocrowns provide greater fracture resistance under axial loading, they are also prone to catastrophic failure.
3. The endocrown marginal design did not significantly impact fracture resistance or failure modes under oblique loading, suggesting no distinct advantage of endocrown designs over conventional crowns.

## Supplemental Information

10.7717/peerj.21801/supp-1 Supplemental Information 1 Raw data of fracture resistance (N) under axial loading - Group TA

10.7717/peerj.21801/supp-2 Supplemental Information 2 Raw data of fracture resistance (N) under axial loading - Group B

10.7717/peerj.21801/supp-3 Supplemental Information 3 Raw data of fracture resistance (N) under axial loading - Group C

10.7717/peerj.21801/supp-4 Supplemental Information 4 Raw data of fracture resistance (N) under axial loading - Group S

## Additional Information and Declarations

### Data Availability

The following information was supplied regarding data availability: The raw data of the fracture resistance (N) under axial loading and oblique loading for all preparation design.
